# Moderate hypofractionated radiotherapy is more effective and safe for localized prostate cancer patients: a meta-analysis

**DOI:** 10.18632/oncotarget.13735

**Published:** 2016-12-01

**Authors:** Ling Cao, Yong-Jing Yang, Zhi-Wen Li, Hong-Fen Wu, Zhu-Chun Yang, Shi-Xin Liu, Ping Wang

**Affiliations:** ^1^ Department of Radiation Oncology, Cancer Hospital of Jilin Province, Changchun 130012, People's Republic of China; ^2^ Department of Anesthesiology, The First Hospital Affiliated to Jilin University, Changchun 130012, People's Republic of China; ^3^ Department of Radiotherapy, Cancer Institute and Hospital of Tianjin Medical University, Tianjin 300060, People's Republic of China

**Keywords:** prostatic neoplasms, hypofractionation, radiotherapy, meta-analysis

## Abstract

To compare the efficacy and safety of moderate hypofractionated radiotherapy (H-RT) with those of conventional radiotherapy (C-RT) in patients with localized prostate cancer, we conducted extensive literature searches of The Web of Science, Embase, Pubmed and Cochrane Library databases. We identified nine studies with 5969 patients for a meta-analysis. We calculated pooled risk ratios (RRs) and the 95% confidence intervals (CIs) for multiple parameters and performed statistical analysis using RevMan 5.3 software. Our analysis showed that the H-RT group obtained greater improvements in the 5-year biochemical or clinical failure-free survival (RR = 1.04, 95% CI:1.01–1.08; *P* = 0.01) and 5-year disease-free survival(RR = 1.04, 95% CI: 1.01–1.07, *P* = 0.02)than the C-RT group. However, the 5-year overall survival rates were comparable in the two groups (RR = 1.02, 95% CI: 0.99–1.04; *P* = 0.18). Comparison of multiple secondary parameters, including grade 2-4 acute/late gastrointestinal toxicity, grade 2–4 acute/late genitourinary toxicity, biochemical failure, local failure, distant failure and prostate cancer-specific mortality between the H-RT and the C-RT groups showed no statistical differences. This meta-analysis thus indicates that in patients with localized prostate cancer, moderate H-RT exerts a great beneficial effect on the primary parameters than C-RT without enhancing adverse events.

## INTRODUCTION

Prostate cancer is the second most common solid tumor diagnosed in older men in the United States, Britain, and Western Europe [1]. The recommended treatment for localized prostate cancer patients is external beam radiotherapy [2]. Although increasing the radiation dose as proposed by the National Comprehensive Cancer Network (NCCN) can improve the control of the disease, increasing the radiation dose using traditional methods can also extend the time of treatment and increase the risk of radiotherapy toxicity [3].

Moderate hypofractionated radiotherapy (H-RT) that uses larger dose radiation treatments (2.2–4 Gy/fraction) has garnered increasing attention compared to conventional radiotherapy (C-RT) (1.8–2.2 Gy/fraction) due to its low α/β value for prostate cancer(approximately 1.4 (0.9–2.2) Gy) [4, 5]. Based on the radiation biology model of prostate cancer, H-RT can improve the treatment without increasing toxicity [6]. Moreover, since H-RT is implemented over a shorter period of time, it is more convenient and cheaper for the patients [7].

In recent years, many RCTs (randomized controlled trials) have focused on using H-RT to treat localized prostate cancer. Some of these studies have shown that moderate H-RT can be more effective for patients with localized prostate cancer without increasing the acute or late toxicities [8]. However, all these trials have not reached consensus in regard to efficacy and safety because they lacked comprehensive evidence [9]. Although two previous studies systematically reviewed and recommended H-RT as the primary management therapy for prostate cancer, their data was not comprehensive as they did not include several phase III randomized prospective trials from recent years [10, 11]. Therefore, the aim of our study was to ascertain the efficacy and toxicity of moderate H-RT in the treatment of localized prostate cancer by conducting an updated systematic review and meta-analysis.

## RESULTS

### Literature survey to identify relevant studies for meta-analysis

**Figure 1:**
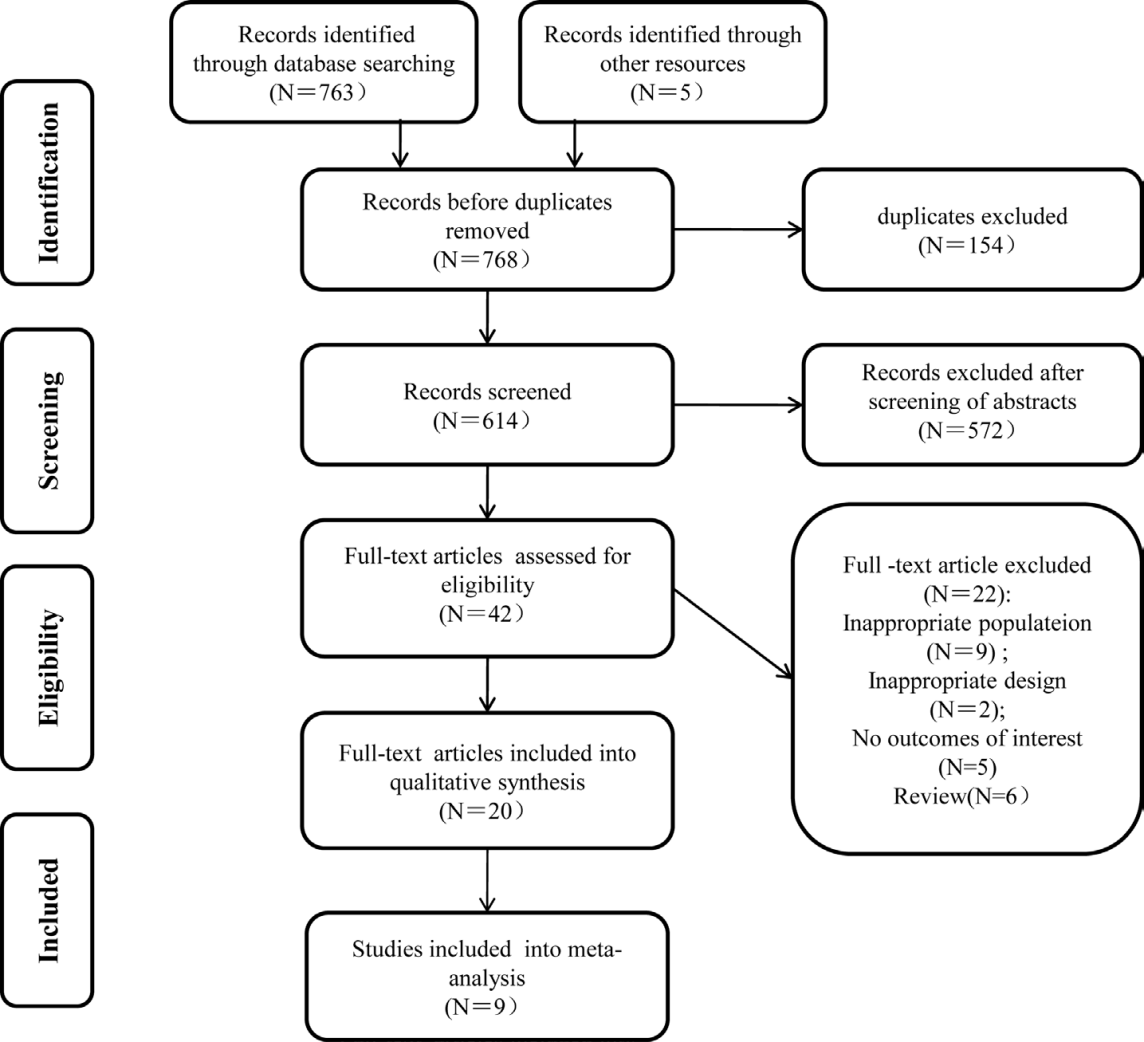
Flow chart of the study selection process

The literature screening process to select studies for our meta-analysis is depicted in Figure 1. A total of 768 references were retrieved from the literature searches, of which 154 records were excluded as duplicates using the “find duplicates” feature of Endnote X7. Further, 572 articles were excluded after screening the titles and abstracts of the references. When the remaining 42 full-text articles were evaluated for eligibility, 20 manuscripts corresponding to 9 studies met the eligibility criteria and were included for our analysis(Lukka2005 [12]; Yeoh 2003, 2006, 2011 [13–15]; Norkus2005, 2009 [16–18]; Arcangeli2009, 2012 [19–21]; Dearnaley2012, 2015, 2016 [22–24]; Hoffman2014 [25]; Pollack2006, 2013 [26, 27]; Aluwini2015, 2016 [28–30]; Lee2016 [31]) (Table 1).

**Table 1:** Baseline characteristics of included trials

Study	Design	*N*	TNM/PSA/ Gleason	RT	Schedule	CTV	PTV	ADT	Median follow-up
Lukka *et.al*[12]	Hypofractionated vs.conventional	466 470	CT1/2N0M0, PSA ≤ 40 ng/ml, Gleason: 2–9	2D	52.5 Gy/20f 66 Gy/33f	Prostate gland alone with a 1.5 cm margin	Margin of 1.5cm in all directions except for 1.0 cm posteriorly	NO	5.7 years
Yeoh *et.al* [13–15]	Hypofractionated vs.conventional	108 109	CT1-2N0M0, PSA < 80 ng/ml, Gleason: 2–10	2D/ 3D-CRT	55 Gy/20f 64 Gy/32f	Prostate gland only	Prostate and the base of seminal vesicles	NO	7.5 years
Norkus *et.al* [16–18]	Hypofractionated vs.conventional	47 44	CT1-3N0M0, PSA ≤ 10 ng/ml, Gleason ≤ 7	3D-CRT	57 Gy/17f 74 Gy/37f	Prostate and the base of seminal vesicles	Margin of 8–10mm in each direction	NO	1 year
Arcangeli *et.al* [19–21]	Hypofractionated vs.conventional	83 85	T2c-4N0M0, PSA > 10 ng/ml, Gleason: 7–10	3D-CRT	62 Gy/20f 80 Gy/40f	Prostate and seminal vesicles	Margin of 1.0 cm in all direction except for 0.6 cm posteriorly	YES	5.8 years
Dearnaley *et.al* [22–24]	Hypofractionated vs.conventional	1074 1065	T1b–3aN0M0, PSA< 30 ng/ml , Gleason ≤ 8	IMRT	60 Gy/20f 74 Gy/37f	Prostate and seminal vesicles+0.5 cm	Margin of 1–1.5 cm in all direction except for 0.5 cm posteriorly	YES	5.2 years
Hoffman *et.al* [25]	Hypofractionated vs.conventional	102 101	T1b-3bN0M0, PSA ≤ 20 ng/ml, Gleason < 10	IMRT	72 Gy/30f 75.6 Gy/42f	Prostate and proximal seminal vesicles	Margin of 1.0 cm in all direction except for 0.4–0.8 cm posteriorly	YES	6.0 years
Pollack *et.al* [26, 27]	Hypofractionated vs.conventional	151 152	T1-3N0M0	IMRT	70.2 Gy/26f 76 Gy/38f	Prostate and proximal seminal vesicles ± pelvic lymph nodes	Conventional: CTV with a margin of 0.7–0.8 cm in all direction except for0.3–0.5 cm posteriorly	YES	5.7 years
Aluwini *et.al* [28–30]	Hypofractionated vs.conventional	410 410	T1b-4N0M0, PSA ≤ 60 ng/ ml	IMRT	64.6 Gy/19f 78 Gy/39f	Prostate ± seminal vesicle	Margin of 0.3–1 cm in each direction	YES	5 years
Lee *et.al* [31]	Hypofractionated vs.conventional	550 542	T1b-2cN0M0, PSA < 10 ng/ml, Gleason: 2–6	3D-CRT/IMRT	70 Gy/28f 73.8 Gy/41f	Prostate gland only	Margin of 0.4–1 cm in each direction	NO	5.8 years

Abbreviations: RT: radiotherapy; vs.: versus; 2D:two-dimensional; 3D-CRT: three-dimensional conformal radiotherapy; ADT: androgen deprivation therapy; IMRT: intensitymodulated radiation therapy; TNM: tumor node metastasis; PSA: prostate-specific androgen; f: fraction.

### Quality analysis of selected studies

**Figure 2:**
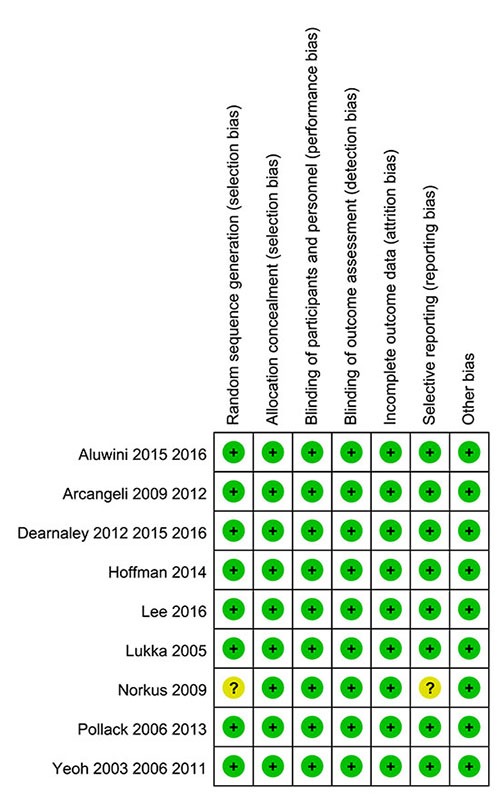
Summary of ‘Risk of bias’: reviewing authors’ judgments regarding risk of bias for every item in each of the included studies

Each of the nine RCTs with 5969 patients that were included in this meta-analysis underwent quality evaluation based on the Handbook of Cochrane for Systematic Reviews of Interventions: “random” was mentioned in all the studies, all the RCTs reported the basic features of patients, and approaches of allocation concealment were reported in all the trails. However, one of the studies did not describe the method of randomized sequence generation and their reasons for incomplete outcome data was interpreted as selective report bias [16–18]. The qualities of the assessed trials in the meta-analysis are shown in Figure 2.

### Analysis of 5-year biochemical or clinical failure free (BCFF)

**Figure 3:**
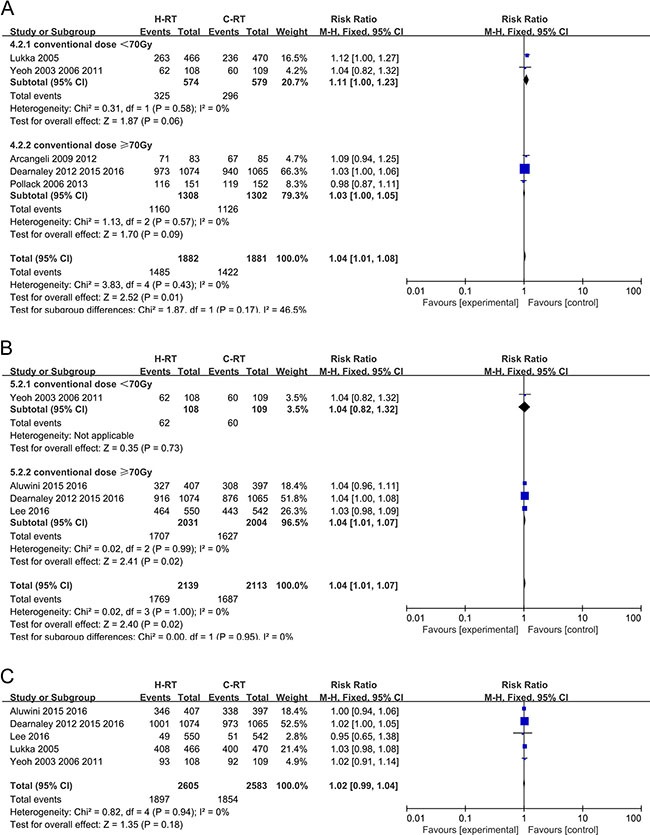
(**A**) Forest plot of risk ratio for 5-year BCFF; (**B**) Forest plot of risk ratio for 5-year DFS; (**C**) Forest plot of risk ratio for 5-year OS.

Five of the nine studies reported the 5-year BCFF rate and included 3763 localized prostate cancer patients in total. Compared to the C-RT group, the moderate H-RT group had increased 5-year BCFF rate (RR = 1.04, 95% CI: 1.01–1.08; *P* = 0.01). Also, fixed-effect model analysis was performed as no statistical heterogeneity was found (Chi^2^ = 3.83, I^2^ = 0%; *P* = 0.43).This benefit was not different between the two subgroups analyzed (conventional group dose < 70 Gy or ≥ 70 Gy), with a negative test for subgroup differences (*P* = 0.17). The complete pooled analysis is shown on Figure 3A.

### Analysis of 5-year disease free survival (DFS)

Four trials with 4252 patients were included in this analysis as they reported the 5-year DFS data. Compared to the C-RT group, the H-RT group showed enhanced 5-year DFS rate (RR = 1.04, 95% CI: 1.01–1.07, *P* = 0.02).The fixed-effect model was used since no statistical heterogeneity was observed (Chi^2^ = 0.02, I^2^ = 0%; *P* = 1.00). Similar to 5 year BCFF, the benefit was similar between the two subgroups analyzed (conventional group dose < 70 Gy or ≥ 70 Gy), with a negative test for subgroup differences (*P* = 0.95). Subgroup analysis indicated that the group with conventional dose ≥ 70 Gy was more beneficial to the patients (*P* = 0.02; Figure 3B)

### Analysis of 5-year overall survival (OS)

Five studies that evaluated 5188 localized prostate cancer patients for OS were included in the analysis. The results of the five trials showed that the 5-year OS did not differ significantly between the H-RT and the C-RT groups (RR = 1.02, 95% CI: 0.99–1.04; *P* = 0.18). This comparison had no statistical heterogeneity (I^2^ = 0%, *P* = 0.94) (Figure 3C).

### Analysis of biochemical failure

**Figure 4:**
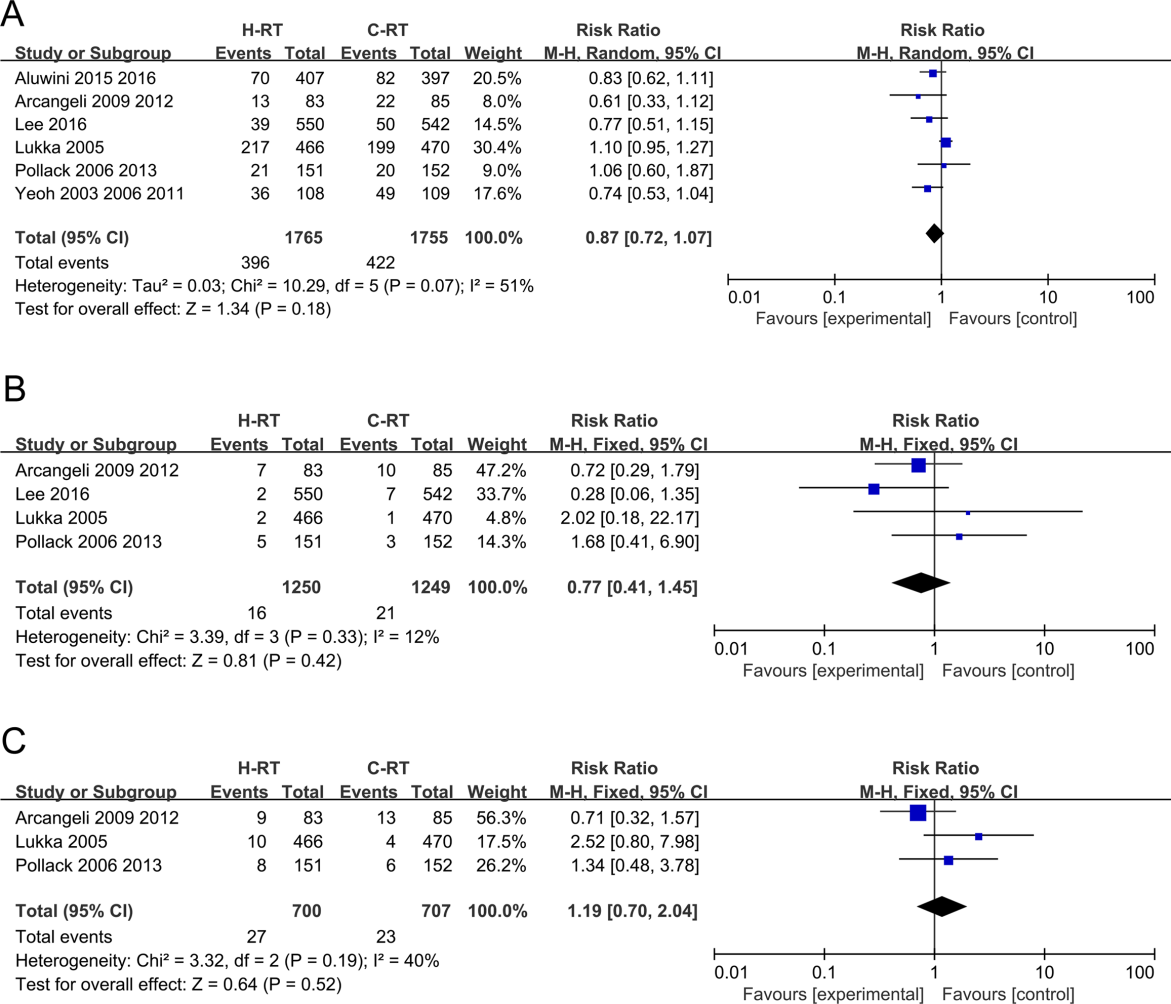
(**A**) Forest plot of risk ratio for biochemical failure; (**B**) Forest plot of risk ratio for local failure; (**C**) Forest plot of risk ratio for distant failure.

Six of the included studies involving 3520 patients with localized prostate cancer reported the results of biochemical failure and were included in the meta-analysis. The C-RT and the H-RT group of patients showed no substantial differences in biochemical failure analysis (RR = 0.87, 95% CI: 0.72–1.07, *P* = 0.18) and showed a high level of heterogeneity based on the random effect model (*P* = 0.07, I^2^ = 51%; Figure 4A).

### Analysis of local recurrence

The outcomes of local recurrence were reported in 4 studies. Our analysis showed that there was no significant benefit for the H-RT group in comparison to the C-RT group (RR = 0.77, 95% CI: 0.41–1.45, *P* =0.42) and they showed a low-level of heterogeneity (*P* = 0.33, I^2^ = 12%; Figure 4B).

### Analysis of distant failure

Three trials that reported distant metastasis outcomes were analyzed by the meta-analysis. No significant difference was detected between the C-RT group and the H-RT group (RR = 1.19, 95% CI: 0.70–2.04, *P* = 0.52). A fixed-effect model showed moderate heterogeneity (*P* = 0.19, I^2^ = 40%; Figure 4C).

### Analysis of grade 2-4 acute gastrointestinal (GI) toxicity

**Figure 5:**
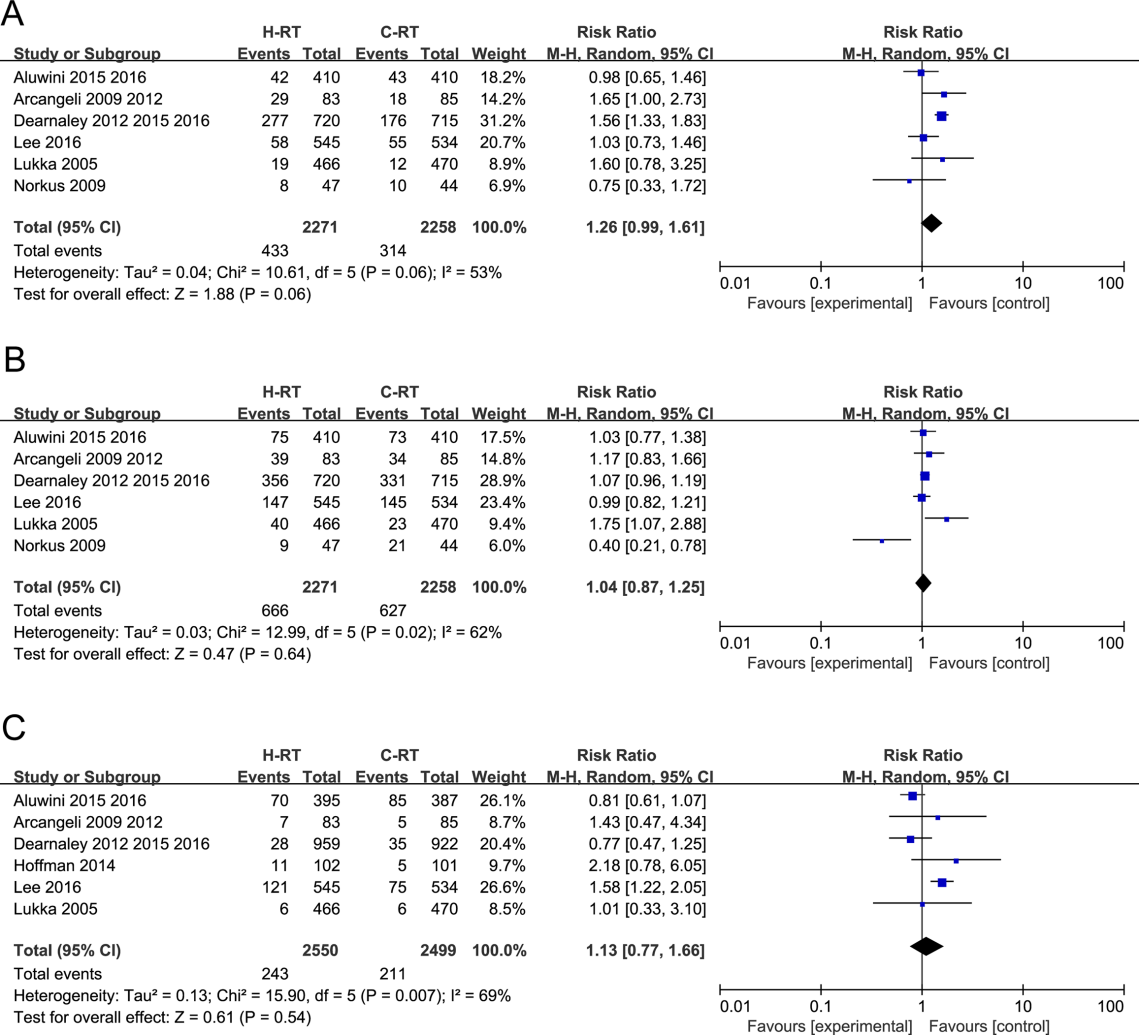
(**A**) Forest plot of risk ratio for Grade 2-4 acute GI toxicity; (**B**) Forest plot of risk ratio for Grade 2-4 acute GU toxicity; (**C**) Forest plot of risk ratio for Grade 2-4 late GI toxicity.

Six trials with 4529 patients that reported the grade 2–4 acute GI toxicity were included in the meta-analysis. The grade 2–4 acute GI toxicity data were similar between the H-RT and C-RT groups (RR = 1.26, 95% CI: 0.99–1.61; *P* = 0.06) (Figure 5A).The random-effect model showed a high level of heterogeneity between the two groups of patients.

### Analysis of grade 2-4 acute genitourinary (GU) toxicity

Six trials with a total of 4529 patients that reported grade 2–4 acute GU toxicity results were included in the meta-analysis. The random-effect model was used due to considerable heterogeneity (Tau^2^ = 0.03; Chi^2^ = 12.99, *P* = 0.02; I^2^ = 62%). We found that the grade 2–4 acute GU toxicity was not significantly increased by H-RT in comparison to C-RT (RR = 1.04, 95% CI: 0.87–1.25; *P* = 0.64; Figure 5B).

### Analysis of grade 2-4 late GI toxicity

Six studies with a total of 5049 patients that reported grade 2–4 late GI toxicity were analyzed by the meta-analysis. The random-effect model showed considerable heterogeneity (Tau^2^ = 0.13; Chi^2^ = 15.90, *P* = 0.007; I^2^ = 69%). We found no significant differences in the grade 2–4 late GI toxicity between the H-RT and the C-RT groups (RR=1.13, 95% CI: 0.77–1.66; *P* = 0.54; Figure 5C).

### Analysis of the grade 2-4 late GU toxicity

**Figure 6:**
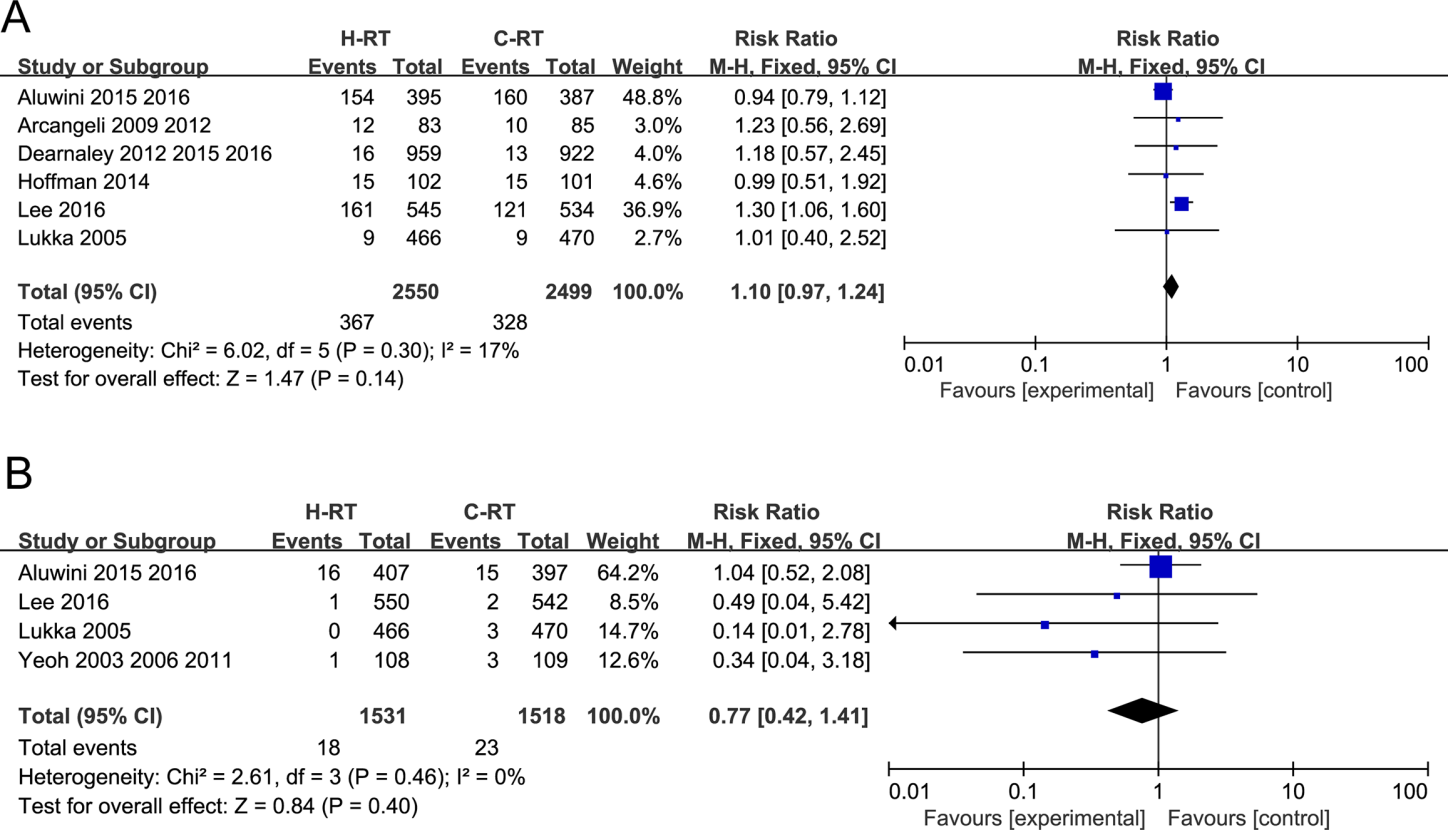
(**A**) Forest plot of risk ratio for Grade 2-4 late GU toxicity; (**B**) Forest plot of risk ratio for specific mortality in prostate cancer.

Six studies reported that included 5049 patients reported the grade 2–4 late GU toxicity results and were analyzed by the meta-analysis. Analysis by the fixed-effect model showed that H-RT did not significantly increase the grade 2–4 late GU toxicity in comparison to the C-RT (RR = 1.10, 95% CI: 0.97–1.24; *P* = 0.14) (Figure 6A).

### Analysis of prostate cancer–specific mortality

Four studies with 3049 patients that reported prostate cancer-specific mortality were included in the meta-analysis. The fixed-effect model was used to calculate the pooled estimates. No significant differences were observed between the H-RT and the C-RT groups (RR = 0.77, 95% CI: 0.42–1.41; *P* = 0.40; Figure 6B).

### Meta-regression analysis

To investigate the effects of various study characteristics on RR estimates, a meta-regression analysis was evaluated for 5-year BCFF and 5-year DFS rates by grouping the studies according to specific characteristics like the trial year, the mode of radiotherapy, clinical stage, and androgen deprivation therapy (ADT). However, the univariate meta-regression analyses did not detect any association between5-year BCFF, 5-year DFS rates and other characteristics (Tables 2 and 3).

**Table 2:** Univariate meta-regression analyses of potential sources of heterogeneity in 5-year BCFF rate

Heterogeneity Factors	Estimate	SE	*Z*-Value	*P*-Value	95% CI	
					LL	UL
**Trial year**						
Univariate	–0.0754	0.0564	−1.3368	0.1813	−0.1861	0.0352
**Mode of radiotherapy**						
Univariate	–0.0706	0.0459	−1.538	0.124	−0.1605	0.0193
**Clinical stage**						
Univariate	0.0531	0.0734	0.7228	0.4698	−0.0908	0.197
**ADT**						
Univariate	−0.0754	0.0564	−1.3368	0.1813	−0.1861	0.0352

Abbreviations and Interpretation: LL = lower limit; SE = standard error; UL = upper limit; trial year ≥ 2012 or < 2012; Mode of radiotherapy: IMRT OR not; clinical stage: T4 OR not; ADT = androgen deprivation therapy; IMRT = intensity-modulated radiation therapy.

**Table 3:** univariate meta-regression analyses of potential sources of heterogeneity in 5-year DFS rate

Heterogeneity Factors	Estimate	SE	*Z*-Value	*P*-Value	95% CI	
					LL	UL
**Trial year**						
Univariate	−0.0072	0.1207	−0.0599	0.9523	−0.2438	0.2293
**Mode of radiotherapy**						
Univariate	0.0038	0.0316	0.12	0.9045	−0.0581	0.0657
**Clinical stage**						
Univariate	0.0001	0.0396	0.003	0.9976	−0.0775	0.0778
**ADT**						
Univariate	0.0038	0.0316	0.12	0.9045	−0.0581	0.0657

Abbreviations and Interpretation: LL = lower limit; SE = standard error; UL = upper limit; trial year ≥ 2012 or < 2012;Mode of radiotherapy: IMRT OR not; clinical stage: T4 OR not; ADT = androgen deprivation therapy; IMRT = intensity-modulated radiation therapy.

## DISCUSSION

Higher doses of radiotherapy have been shown to be more effective in controlling localized prostate cancer [32, 33]. This was confirmed by a meta-analysis that included seven RCTs with a total of 2812 patients [34].This study showed that higher doses of radiation were superior to the conventional dose in low-,intermediate-, and high-risk prostate cancer patients and proposed that higher doses of radiotherapy should be offered as a treatment option for all prostate cancer patients regardless of their risk classification. The 2014 NCCN Guidelines recommended a minimum radiation dose of 75.6–79.2 Gy for low-risk patients, and 81 Gy for intermediate- and high-risk patients [35]. However, increasing the external radiation dose without enhancing the single radiation dose would result in increased frequency of therapy that would increase the duration of treatment and cost to the patients [8].

Recently, moderate hypofractionated external beam radiotherapy has gained popularity in the treatment of prostate cancer. Evidence has shown that prostate cancer has a lower α/β ratio compared to the surrounding organs at risk and hence, there is a potential clinical benefit in using moderate hypofractionated radiotherapy [4]. In addition, moderate H-RT has numerous additional advantages, such as reducing treatment time, medical resources, and improving the patient's convenience [5]. Although several trials have demonstrated the safety and efficacy of moderate H-RT compared to C-RT in regard to localized prostate cancer, the outcomes have been inconsistent [9]. Therefore, we undertook this meta-analysis to further evaluate the therapeutic efficacy and safety of moderate H-RT.

We found that the moderate H-RT groups had significantly increased 5-year BCFF (RR = 1.04, 95% CI:1.01–1.08; *P* = 0.01) and 5-year DFS rates (RR = 1.04, 95% CI: 1.01–1.07, *P* = 0.02). Apart from the efficacy we focused on the adverse events of moderate H-RT in localized prostate cancer. In this meta-analysis, most of the included studies did not detect any increase in the grade 2–4 acute/late toxicity in H-RT. Pollack and others had reported that the patients with preexisting urinary dysfunction could have increased GU toxicity in the moderate H-RT group [27]. Lee and others showed that adverse late grade 2 and 3 GI and GU events increased (HR:1.31 to 1.59) in patients who were treated with H-RT [31]. However, our pooled analysis showed no significant increase in the grade 2–4 acute/late gastrointestinal toxicity and grade 2–4 acute/late genitourinary toxicity. Therefore, we concluded that moderate H-RT in localized prostate cancer was superior to conventional radiotherapy and did not elicit increased toxicity.

In 2013, a meta-analysis study found that freedom from biochemical failure was similar in patients receiving either moderate hypofractionated or conventional radiotherapy [10]. Although the incidence of acute adverse gastrointestinal events was higher in the hypofractionated group, acute genitourinary toxicity and other late adverse events were similar in both groups. In 2015, another meta-analysis performed by Koontz and colleagues found similar biochemical control and late grade 2 genitourinary and gastrointestinal toxicities between moderate hypofractionated and conventional radiotherapy [11]. The outcomes of our study are not consistent with these two previous reports because these studies had limited data and did not include the results from several recently published phase III clinical trials, including the CHHiP [24], HYPRO [30] and RTOG-0415 [31] trials. Moreover, besides the aforementioned outcome measures, our meta-analysis included a total of eleven indicators and provided a thorough comparison between the moderate H-RT group and C-RT group regarding differences in efficacy and toxicities, thus providing better evidence for the clinicians regarding the choice of radiotherapy. However, since the studies analyzed by this meta-study had varying hypofractionation schemes, our study could not determine the optimal hypofractionation scheme, thus limiting our findings.

In conclusion, we present systematic review and meta-analysis showing that moderate hypofractionated radiotherapy could significantly improve the therapeutic effect in patients suffering from localized prostate cancer without increasing the acute or late toxicities to the gastrointestinal or genitourinary system. However, our results should be interpreted with caution due to insufficient information size and need to be addressed by larger RCTs.

## MATERIALS AND METHODS

### Study selection criteria

To compare conventional radiotherapy (with doses per fraction from 1.8 to 2.2 Gy) versus moderate hypofractionated (2.2 to 4 Gy), RCTs with a parallel design with patients suffering from localized prostate cancer without metastases were included. Studies with quasi-randomized, single-arm phase II or non-original, non-randomized trials were excluded from the analysis.

The primary endpoints were 5-year biochemical or clinical failure-free (BCFF) and 5-year disease-free survival (DFS) rates, while secondary measures of outcome included 5-year overall survival (OS),biochemical failure(BF),local failure, distant failure, grade 2–4 acute gastrointestinal(GI)toxicity, grade 2–4 acute genitourinary (GU)toxicity, grade 2–4 late GI toxicity, grade 2–4 late GU toxicity and prostate cancer-specific mortality.

### Literature search

Relevant articles for the meta-analysis were identified through searches in the Embase, Pubmed, Cochrane Library and Web of Science databases until June16, 2016. There was no limitation in the electronic literature search regarding the publication year, status or language in the electronic search. The searches were conducted using either Emtree or MeSH terms and were appropriately adjusted in various electronic records. In order to check the potentially qualifying articles, abstracts from the academic meetings mentioned below were included. Besides the electronic search for original papers, a review of the references was conducted.

### Data extraction and assessment of the risk of bias

Two independent investigators conducted the literature search and tested its quality. The bias risk of the included studies was evaluated according to the Handbook of the Cochrane for Systematic Reviews of Interventions [36]. A third reviewer was responsible for addressing any disagreements between the original two independent investigators. The studies were classified into unclear, low or high bias risk groups based on the evaluation on the general sequence allocation, allocation concealment, blinding of personnel and participants(performance bias), outcome evaluation blinding (detection bias), partial addressing of the data, presence of biases in the reports and other bias sources that could influence the validity of the research.

### Statistical analysis

Statistical analysis was conducted using the RevMan 5.3 software (Nordic Cochran Centre in Copenhagen of Denmark, 2014).Meta-regression analysis was performed with R version 3.3.1 (2016-06-21)and “meta” package. The risk ratio (RR) was assessed and the 95% confidence intervals (CI) were calculated to evaluate the various parameters. Shared heterogeneity among the studies was evaluated using the I^2^ and Chi^2^ tests. We used the fixed-effect model when the data lacked heterogeneity (*P* > 0.10, I^2^ < 50%). Otherwise, the random-effect model was used. Three potential sources of heterogeneity, including statistical, clinical and methodological, were studied. The I^2^ approach was used to measure heterogeneity and values > 50% were regarded as high level of heterogeneity, 25–50% as moderate level and below 25% as low level [12, 37]. We also explored heterogeneity through sensitivity analysis by conducting subgroup analysis. If excessive heterogeneity occurred, descriptive analysis was employed to conduct the meta-analysis. Meta-regression analysis was conducted to determine the possible causes of heterogeneity and to identify the influence of the various exclusion criteria on the overall risk estimate. A *P-value* < 0.05 was considered statistically significant [38].

## SUPPLEMENTARY MATERIALS AND METHODS


