# Supplementary material for: Moderate hypofractionated radiotherapy is more effective and safe for localized prostate cancer patients: a meta-analysis
Source: Oncotarget. 2016 Dec 1;8(2):2647–58. doi: 10.18632/oncotarget.13735 (PMC5356830; doi:10.18632/oncotarget.13735)
Supplement: Supplementary file 1 [file oncotarget-08-2647-s001.pdf]

Moderate hypofractionated radiotherapy is more effective and safe for localized prostate cancer patients: a meta-analysis

Supplementary Materials

Appendix: Pubmed search terms

#1: (((((((((((((((((Prostate Neoplasms [Title/Abstract]) OR Neoplasms, Prostate [Title/Abstract]) OR Neoplasm, Prostate [Title/Abstract]) OR Prostate Neoplasm [Title/Abstract]) OR Neoplasms, Prostatic [Title/Abstract]) OR Neoplasm, Prostatic [Title/Abstract]) OR Prostatic Neoplasm [Title/Abstract]) OR Prostate Cancer [Title/Abstract]) OR Cancer, Prostate [Title/Abstract]) OR Cancers, Prostate [Title/Abstract]) OR Prostate Cancers [Title/Abstract]) OR Cancer of the Prostate [Title/Abstract]) OR Prostatic Cancer [Title/Abstract]) OR Cancer, Prostatic [Title/Abstract]) OR Cancers, Prostatic [Title/Abstract]) OR Prostatic Cancers [Title/Abstract]) OR Cancer of Prostate [Title/Abstract])) OR "Prostatic Neoplasms" [Mesh]
#2: (((((((Radiotherapy [Title/Abstract]) OR Radiotherapies [Title/Abstract]) OR Radiotherapy, Targeted [Title/Abstract]) OR Radiotherapies, Targeted [Title/Abstract]) OR Targeted Radiotherapies [Title/Abstract]) OR Targeted Radiotherapy [Title/Abstract])) OR «Radiotherapy» [Mesh]
#3: (((((((((((Hypofractionation, Dose [Title/Abstract]) OR Radiotherapy Minibeams [Title/Abstract]) OR Minibeam, Radiotherapy [Title/Abstract]) OR Minibeams, Radiotherapy [Title/Abstract]) OR Radiotherapy Minibeam [Title/Abstract]) OR Hypofractionated Dose [Title/Abstract]) OR Dose, Hypofractionated [Title/Abstract]) OR Doses, Hypofractionated [Title/Abstract]) OR Hypofractionated Doses [Title/Abstract]) OR Hypofractionated [Title/Abstract]) OR Hypofractionation [Title/Abstract])) OR "Dose Hypofractionation" [Mesh]
#4: (((((Randomized controlled trial [Title/Abstract]) OR controlled clinical trial [Title/Abstract]) OR randomized [Title/Abstract]) OR placebo [Title/Abstract]) OR randomly [Title/Abstract]) OR trial [Title/Abstract]
#5: #1 AND #2 AND #3 AND #4
